# Glaucomatous optic nerve damage in the contralateral eye of a patient with peripapillary retinoschisis: a case report

**DOI:** 10.1186/s12886-023-02887-4

**Published:** 2023-04-03

**Authors:** Wenbo Zhang, Tian Tian, Liu Yang

**Affiliations:** Department of Ophthalmology, Peking University First Hospital, No. 8 Xishiku Street, Xicheng District, Beijing, 100034 China

**Keywords:** Peripapillary retinoschisis, Glaucoma, Retinal nerve fiber layer defect, Case report

## Abstract

**Background:**

Peripapillary retinoschisis (PPRS) is often associated with glaucomatous eyes. It usually occurs in eyes with a more advanced stage of glaucoma with obvious optic nerve damage. We report a patient who was found to have PPRS in one eye during a routine physical examination without obvious glaucoma symptoms. Further examination revealed glaucomatous visual field loss and retinal nerve fiber layer defects in the contralateral eye.

**Case presentation:**

A 55-year-old man presented for a routine physical examination. The anterior segment was normal in both eyes. Fundus examination revealed an elevated and red optic disc in the right eye. In addition, scattered patchy red lesions were seen on the retina on the temporal side of the optic disc. The color and boundary of the left optic disc were normal, and the cup-to-disc ratio was 0.6. Optical coherence tomography showed retinoschisis on the optic nerve head of the right eye throughout the entire circumference, extending to the retina on the temporal side of the optic disc. The intraocular pressure was 18 mmHg OD and 19 mmHg OS. The patient was diagnosed with PPRS (OD). However, no optic disc pit or optic disc coloboma was found. Further examination showed that the visual field of the patient’s right eye was generally normal, while a glaucomatous visual field defect was found in the left eye, which manifested as a nasal step visual field defect. Moreover, stereophotography and a red-free fundus image revealed two retinal nerve fiber layer defects in the supratemporal and infratemporal regions of the retina of the left eye. Continuous intraocular pressure measurement found that the intraocular pressure fluctuated between 18 and 22 mmHg OD and 19–26 mmHg OS during the daytime. Primary open-angle glaucoma was then diagnosed.

**Conclusions:**

In this case, we found that PPRS was associated with glaucomatous optic nerve changes and visual field defects in the fellow eye.

## Background

Peripapillary retinoschisis (PPRS) is the splitting of the retina around the optic nerve head (ONH) and often occurs in patients with congenital optic disc abnormalities, such as optic disc pit and optic disc coloboma [1–3]. Recent evidence indicates that PPRS is more likely to develop in glaucoma patients than in normal people [4–11]. PPRS usually occurs in eyes with a more advanced stage of glaucoma, a higher intraocular pressure, more severe optic nerve damage, a poorer visual field and a thinner retinal nerve fiber layer (RNFL) [4, 6, 9, 10, 12, 13]. However, the patient we reported was found to have PPRS in one eye during a routine physical examination without obvious glaucoma symptoms. Further examination revealed glaucomatous visual field defects and RNFL defects in the contralateral eye.

## Case presentation

**Fig. 1:**
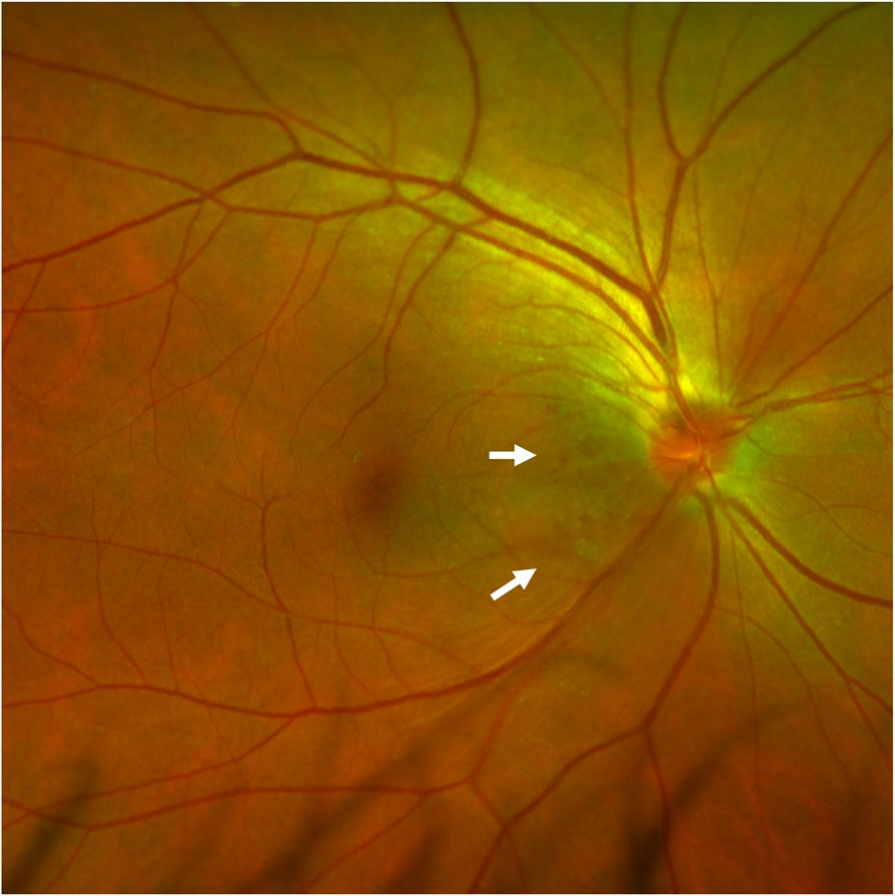
Fundus photography of the right eye. The optic nerve head is elevated, red and boundary-blurred. Scattered patchy red lesions are seen on the retina on the temporal side of the optic disc (marked with arrows)

**Fig. 2:**
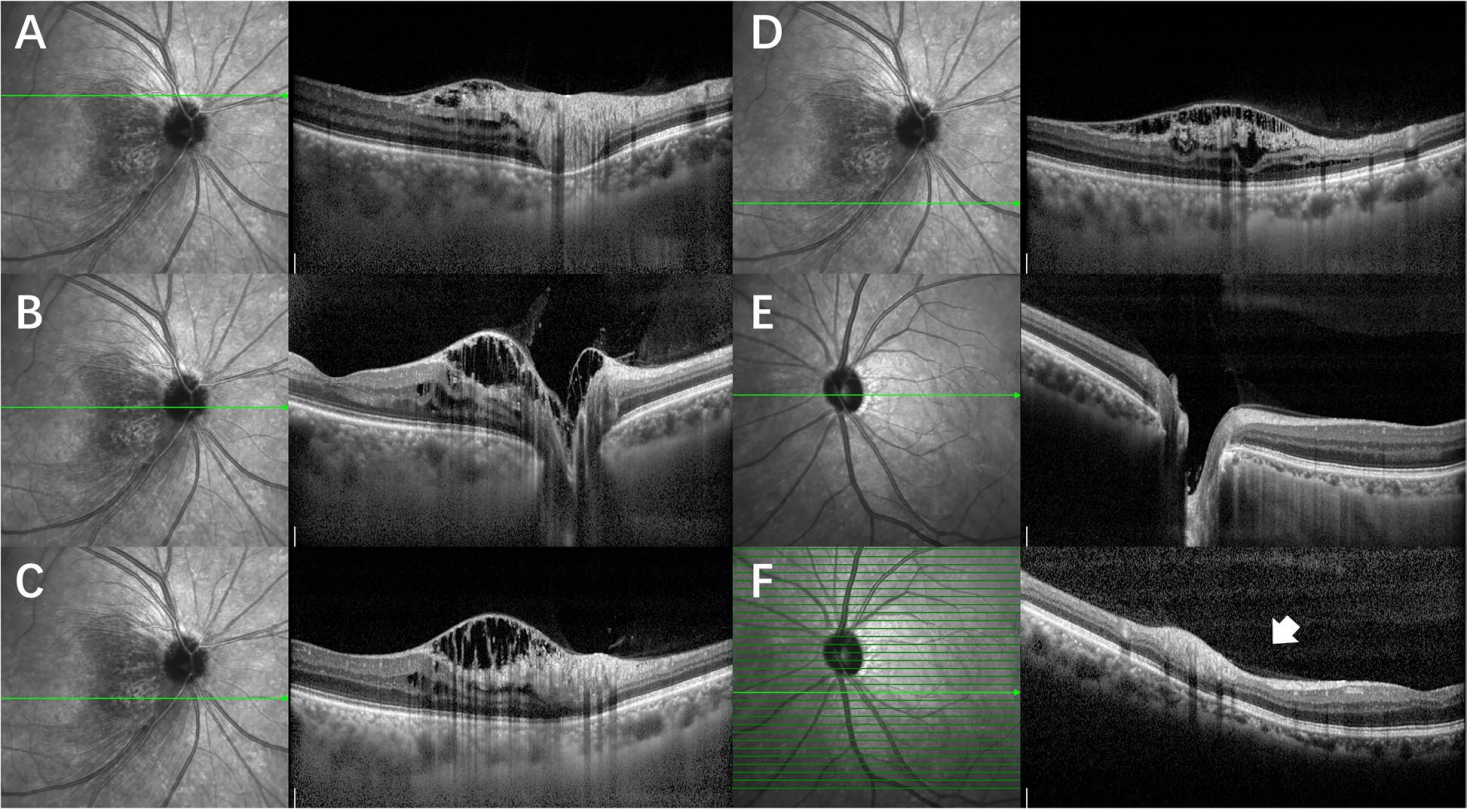
Optical coherence tomography (OCT) and infrared fundus image of the optic disc of both eyes. OCT shows retinoschisis on the optic nerve head of the right eye throughout the entire circumference, extending to the temporal retina. The splitting is located mainly in the retinal nerve fiber layer, ganglion cell layer, inner nuclear layer and outer nuclear layer. Infrared fundus image shows the lesion on the temporal side of the optic nerve head of the right eye presents as a wedge-shaped dark area with the base attached to the edge of the optic disc, scattered along the nerve fiber bundles (**A**, **B**, **C**, **D**). The OCT image and infrared fundus image of the left eye is normal (**E**). OCT shows obvious retinal nerve fiber layer thinning through the areas of retinal nerve fiber layer defects in the left eye (marked with an arrow, **F**)

**Fig. 3:**
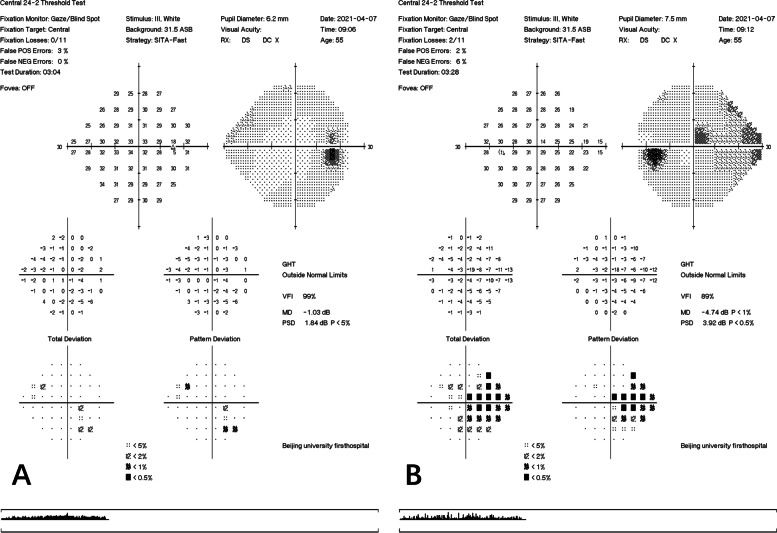
Automated perimetry examination of both eyes. The right visual field is generally normal (**A**), and the left visual field shows nasal step visual field defects (**B**)

**Fig. 4:**
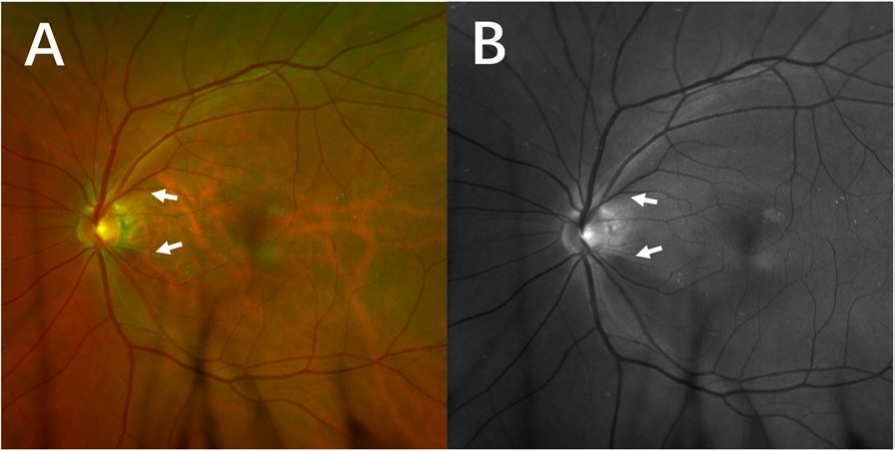
Stereophotography and red-free fundus image of the left eye. The color and boundary of the left optic disc are normal, and the cup-to-disc ratio is 0.6. Retinal nerve fiber layer defects are found in the supratemporal and infratemporal regions of the retina (marked with arrows, **A**). Red-free fundus image shows obvious retinal nerve fiber layer defects in the supratemporal and infratemporal regions of the retina (marked with arrows, **B**)

A 55-year-old man presented for a routine physical examination. His best-corrected visual acuity was 20/20 in the right eye (0 Diopters Sphere) and 20/20 in the left eye (-2.00 Diopters Sphere), and intraocular pressure was 18 mmHg OD and 19 mmHg OS (central corneal thickness: 539 µm OD and 546 µm OS). In slit-lamp biomicroscopy examination, the cornea, anterior chamber, iris and lens were normal. However, fundus examination (Optos p200t, Optos PLC, Dunfermline, UK) revealed an elevated, red, boundary-blurred ONH in the right eye. In addition, scattered patchy red lesions were seen on the retina on the temporal side of the optic disc, similar to retinal hemorrhage (Fig. 1, pseudocolor). The color and boundary of the left optic disc were normal, and the cup-to-disc ratio was 0.6. Spectral-domain optical coherence tomography (OCT, Spectralis HRA-OCT, Heidelberg Engineering, Heidelberg, Germany) was performed and showed retinoschisis on the ONH of the right eye throughout the entire circumference, extending to the retina on the temporal side of the optic disc. The splitting was mainly located in the RNFL layer. The outer nuclear layer, inner nuclear layer and ganglion cell layer were also involved in the temporal side of the optic disc. The red elevated ONH was caused by the splitting and towering of the neuroretina rim. On infrared fundus imaging, the lesion on the temporal side of the optic disc presented as a wedge-shaped dark area with the base attached to the edge of the optic disc, scattered along the nerve fiber bundles (Fig. 2A-D). The OCT and infrared fundus image of the left eye was normal (Fig. 2E). The patient was diagnosed with PPRS (OD). However, no optic disc pit or optic disc coloboma was found. Since PPRS is often associated with glaucoma, visual field examination was subsequently performed. Visual field examination (Humphrey visual field analyzer 750i, Carl Zeiss Meditec, Dublin, USA) showed that the right side was generally normal, while a nasal step visual field defect was found in the left eye (Fig. 3). Furthermore, stereophotography and a red-free fundus image revealed two RNFL defects in the supratemporal and infratemporal regions of the retina of the left eye (Fig. 4). In line scan OCT, obvious RNFL thinning was found through the areas of RNFL defects (Fig. 2F). Intraocular pressure was continuously measured and fluctuations between 18 and 22 mmHg OD and 19–26 mmHg OS were found during the daytime. Because no other factors leading to secondary glaucoma were found and the anterior chamber angle was open, the patient was diagnosed with primary open-angle glaucoma and treated with local intraocular pressure lowering drugs.

## Discussion and Conclusions

At present, more studies support that PPRS tends to occur in glaucoma patients. The incidence of PPRS in glaucoma patients is approximately 3.1-6.0%, which is significantly higher than the incidence of approximately 0.5% in normal people [4–6]. PPRS can be found in various types of glaucoma, such as open-angle glaucoma [7, 8], narrow-angle glaucoma [9], secondary glaucoma [10], and even normal-tension glaucoma [11].

The specific mechanism of PPRS in glaucoma is still controversial. However, there are several possibilities. Kahook et al. suggested that the fluctuation in the intraocular pressure accompanied by small changes in the axial length is the main cause of PPRS [9]. Lee et al. reported that the liquid flows into the ONH from the subarachnoid space and vitreous cavity through the lamina cribrosa defect, thus causing PPRS. Glaucoma patients are more likely to have lamina cribrosa defects coupled with the destruction of glial barrier function caused by atrophy of inner nerve retinal tissue, which aggravates the entry of liquid into the retina[3]. Other possible mechanisms include lateral mechanical forces on the ONH caused by reactive gliosis of Müller cells [1, 6] and vitreous traction [11, 14].

PPRS usually resolves spontaneously [8], but it is still worthy of attention. First, spontaneous recovery of PPRS can lead to a sudden decrease in RNFL thickness measurements, resulting in misinterpretation of the rapid progression of glaucoma [7, 8]. Second, recent studies have shown that the emergence of PPRS is indeed related to the rapid aggravation of glaucoma [1, 6]. Third, based on this case, we found that PPRS can sometimes indicate the presence of glaucoma.

This patient was found to have PPRS in his right eye during a routine physical examination. No history of glaucoma or obvious symptoms was revealed. OCT showed no signs of optic disc pit or lamina cribrosa defect. Due to the close relationship between PPRS and glaucoma, we performed relevant examinations. Further examinations found that the patient actually had high intraocular pressure and glaucomatous optic nerve damage. This suggests that PPRS may indicate the existence of glaucoma, and additional examination should be performed for diagnosis. Interestingly, in this patient, glaucomatous optic nerve damage was found in the contralateral eye rather than the eye with PPRS. In fact, PPRS often occurs in a more advanced stage of glaucoma, and the affected eye is often accompanied by higher intraocular pressure, larger intraocular pressure fluctuation, higher cup-to-disc ratio, thinner RNFL thickness, and poorer visual field [4, 6, 9, 10, 12, 13]. In addition, PPRS often appears at the site of RNFL defects, and the range and location of PPRS often overlap with those of RNFL defects [1, 4–6, 15, 16]. However, we found a high intraocular pressure, a nasal step visual field defect and two RNFL defects in the contralateral eye with PPRS. Therefore, although PPRS is associated with glaucomatous eyes, it can appear in the eye without obvious glaucomatous optic nerve damage. Visual field loss and RNFL defects can be found in the contralateral eye.

However, the above view is still speculative and requires more evidence to be confirmed. In fact, even if no visible disc pit was found in this case, occult disc pits may still exist. In Hedels C’s study, small hyporeflective cavitations were found adjacent to the ONH in a patient with PPRS without an obvious optic pit using OCT with an enhanced depth imaging model [17]. Nagesha CK et al. also found cavitation in the disc stroma in OCT images of a glaucoma patient with PPRS without visible disc pits, demonstrating an occult disc pit [18]. Using a narrow band raster OCT scan, Banerjee M et al. detected cavitations along the slope of the ONH in a patient with a circumscribed, hypopigmented macular lesion with peripapillary extension and no clinical evidence of disc pits [19]. In our study, we did not find such a sign of cavitation in the ONH on OCT images. Nevertheless, because there are gaps between lines in the OCT line scan, some lesions may be missed, so the possibility of occult disc pit cannot be completely ruled out.

In general, PPRS does not require additional treatment and can resolve spontaneously within an average of 9 months [8]. Controlling intraocular pressure can also alleviate PPRS, whether through trabeculectomy or drug administration [4, 14]. Unfortunately, this patient was not followed up long enough to observe the outcome of PPRS.

This case report has some limitations in regards to the clinical data collection. First, we did not repeat the visual field examination for the patient, which may affect the reliability of the results. Second, ONH scan OCT, which can display RNFL thickness in different areas around the ONH, has not been performed. However, in line scan OCT, we found obvious RNFL thinning through the areas of RNFL defects. Third, we did not measure the diurnal variation in intraocular pressure for this patient.

In conclusion, we report a patient who was found to have PPRS in one eye without obvious glaucoma symptoms. Further examination revealed glaucomatous optic nerve damage in the contralateral eye. PPRS may indicate the existence of glaucoma, further examination and follow-up is required. In this case, we found that PPRS was associated with glaucomatous optic nerve changes and visual field defects in the fellow eye.

## Data Availability

Not applicable.

## Abbreviations

OCT: Optical coherence tomography
ONH: Optic nerve head
PPRS: Peripapillary retinoschisis
RNFL: Retinal nerve fiber layer.
